# An Update on Psychopharmacological Treatment of Autism Spectrum Disorder

**DOI:** 10.1007/s13311-022-01183-1

**Published:** 2022-01-14

**Authors:** Ramkumar Aishworiya, Tatiana Valica, Randi Hagerman, Bibiana Restrepo

**Affiliations:** 1 Medical Investigation of Neurodevelopmental Disorders (MIND) Institute, University of California Davis, 2825 50th Street, Sacramento, CA 95817 USA; 2 Khoo Teck Puat-National University Children’s Medical Institute, National University Health System, 5 Lower Kent Ridge Road, Singapore, 119074 Singapore; 3 Department of Pediatrics, Yong Loo Lin School of Medicine, National University of Singapore, 1E Kent Ridge Road, Singapore, 119228 Singapore; 4 Association for Children With Autism, Chisinau, Moldova; 5 Department of Pediatrics, University of California Davis School of Medicine, 4610 X St, Sacramento, CA 95817 USA

**Keywords:** Autism spectrum disorder, Autism, Pharmacology, Medications

## Abstract

While behavioral interventions remain the mainstay of treatment of autism spectrum disorder (ASD), several potential targeted treatments addressing the underlying neurophysiology of ASD have emerged in the last few years. These are promising for the potential to, in future, become part of the mainstay treatment in addressing the core symptoms of ASD. Although it is likely that the development of future targeted treatments will be influenced by the underlying heterogeneity in etiology, associated genetic mechanisms influencing ASD are likely to be the first targets of treatments and even gene therapy in the future for ASD. In this article, we provide a review of current psychopharmacological treatment in ASD including those used to address common comorbidities of the condition and upcoming new targeted approaches in autism management. Medications including metformin, arbaclofen, cannabidiol, oxytocin, bumetanide, lovastatin, trofinetide, and dietary supplements including sulforophane and N-acetylcysteine are discussed. Commonly used medications to address the comorbidities associated with ASD including atypical antipsychotics, serotoninergic agents, alpha-2 agonists, and stimulant medications are also reviewed. Targeted treatments in Fragile X syndrome (FXS), the most common genetic disorder leading to ASD, provide a model for new treatments that may be helpful for other forms of ASD.

**Supplementary Information:**

The online version contains supplementary material available at 10.1007/s13311-022-01183-1.

## Introduction

ASD is a complex neurodevelopmental, biologically based condition with an estimated prevalence of 1 in 44 people [1] that impacts all areas of child development — from behavior, problem solving abilities and self-care skills, to complex social communication ability, language, and executive functioning skills. The range of symptoms and severity of ASD vary greatly from child to child, and clinical manifestations depend on the individual’s age, cognitive and language abilities, and co-occurring conditions. The last revision of the Diagnostic and Statistical Manual (DSM-5) defines ASD as impairments in two main domains: (1) social communication and interaction, which comprises challenges in social-emotional reciprocity, challenges in using nonverbal strategies during social interaction, and challenges developing, maintaining and understanding relationships, and (2) restricted, repetitive, and stereotyped patterns of behavior, manifested by unusual repetitive movements or behaviors, restricted interests, insistence on sameness and inflexible adherence to routines, as well as sensory challenges ranging from seeking to avoiding certain sensory stimuli [2–4]. However, a range of behavioral, cognitive, and emotional disturbances in ASD can also be attributed to a high rate of co-occurring mental health and medical conditions such as attention deficit hyperactivity disorder (ADHD), anxiety, depression, phobias, intellectual disability, speech/language impairment, restrictive/avoidant food intake, sleep issues, sensory processing issues, and genetic conditions. This often makes the recognition, diagnosis, and clinical management of ASD even more complex and difficult [5–8].

Classic medical management of medical conditions has largely revolved around pharmacological treatment. However, despite decades of research in ASD, current evidence has only established behavioral (non-pharmacological) treatments as the mainstay of management to address the core symptoms of ASD. Part of the reason for a lack of efficacy in many treatment studies stems from the heterogeneous etiology underlying the overall term of ASD. Some studies have subdivided enrolled patients either by their genetic etiology or phenotypic features to address this. The aim of this paper is to provide a current update on the pharmacological treatments available for ASD and therapeutic subtypes of ASD, covering both the established ones and upcoming/emerging treatments which have potential based on scientific evidence to become standard treatments in the next few years. A systematic literature search was completed on Medline, Scopus, and Embase with key search terms of “autism,” “autism spectrum disorder,” “targeted treatments,” “pharmacological therapy,” and “management” to identify relevant articles. Here we have highlighted the psychopharmacological treatments that have the most efficacy and are also, in most cases, available now or in the near future to clinicians. However, we also recognize the current mainstay of behavioral intervention in the management of ASD and will briefly review those which are supported by strong empirical evidence.

## Non-pharmacological (Behavioral) Interventions

In 1987, Lovaas published an article which introduced a new treatment approach describing a significant improvement of IQ scores and educational functioning in almost 50% of children with ASD [9]. Also known as The Lovaas Method of Applied Behavior Analysis, and subsequently as discrete trial training (DTT), it is an intensive, highly structured, long-term, one-on-one behavior intervention designed for young children, which has a strong empirical support and has become the foundation for many of the evidence-based behavioral interventions in use today [10]. Subsequently, through decades of extensive research, a number of modifications and adaptations of the Lovaas method have since been developed. These can be used in different settings, environments, and procedures, and have been shown to be effective in addressing the core impairments of ASD in social communication, speech, behaviors, play, and learning [11–14].

Odom et al. [13] and Wong et al. [15] have classified behavioral evidence–based interventions into two groups: comprehensive treatment models (CTMs) and focused interventions.

Comprehensive treatment models focused on core ASD symptoms have been found to improve language, cognitive, and functional language skills in young children, using intensive and long-term multi-disciplinary strategies in naturalistic environments. Instructions can be provided at home or in a classroom setting, individually or in a group, provided by instructors or by parents. Examples of well-established CTMs include Early Behavior Intervention (EIBI) [16], Early Start Denver model (ESDM) [17], Developmental, Individual difference, relationship-based model (DIR/Floortime, or Greenspan model) [18], Pivotal Response Training (PRT) [19], and Treatment and education of autistic and related communication handicapped children (TEACCH) [20].

Focused interventions address a single skill or a specific area of developmental domain and are provided for a short time, until the skill is mastered. They can also be effective to address life-threatening or socially inappropriate behaviors that require rapid addressing. Examples include social skill training, toilet training, modeling, cognitive behavioral intervention, and behavioral strategies like prompting, ignoring, time delay, reinforcement, discrete trial teaching, and extinction. These can be implemented as a structured session or in a naturalistic setting at home, school, clinic, or community settings, with peers or parents, and have behavioral, developmental, or educational purposes. Peer-mediated Instruction and Intervention (PMII), also known as “Peer Modeling,” “Peer Initiation Training,” “Peer support” [21, 22], and Picture Exchange Communication System (PECS) [23], are also other examples of focused interventions.

Behavioral interventions work most effectively when started at an early age and the majority cater to young children to optimize their development and learning skills. The sociocultural beliefs and economic capability of the family also moderate treatment impact and outcome [24]. However, behavioral interventions do have a role in older children, adolescents, and adults as well; the targets of these interventions change in older individuals to include social, vocational, leisure skills, and independent living. Research in behavioral interventions for adults with ASD is still limited and will need to be expanded in future. 

## Established Psychopharmacological Treatments

The use of psychotropic medications has markedly increased over the last decades; approximately two-thirds of autistic adolescents have been treated with psychotropic medications, especially those with challenging behaviors and co-occurring conditions like intellectual disability (ID), medical, and mental health diagnoses. Co-occurring mental health conditions have been reported in approximately 70% of autistic individuals ranging from attention deficit and hyperactivity disorder (ADHD), irritability, aggression, mood, and anxiety issues [8, 25, 26]. Mandell et al. reported that 56% were prescribed at least one psychotropic medication and 20% were prescribed three or more [27]. Individuals with ASD frequently are treated with multiple medications, including off-label use (e.g., use of antipsychotic medications in younger children). Studies have reported high rates of polypharmacy ranging from 12 to 35% based on the type of studies [28, 29]. The increasing prescription rates for individuals with ASD is not completely understood. For instance, some authors have postulated that this may be influenced by improvements in diagnostic and clinician awareness of co-occurring mental health issues [25]. However, other researchers have reported demographic factors influencing pharmacological treatment. For instance, in a large study, those who were uninsured or exclusively privately insured were less likely to use more than 3 medications than were those insured by Medicaid [30]. Prescription medications may also be affected by demographic factors including race, ethnicity, and geography. Studies have reported that challenging behaviors and mental health diagnoses are influencing factors [29]. For example, polypharmacy is often necessary since one treatment for anxiety may not be helpful for another comorbid condition such as ADHD. Such polypharmacy will be more common as specific treatments for dysfunctional pathways are utilized which go hand in hand with other treatments for common comorbidities of ASD. An example is metformin which can downregulate the mTOR pathway, and this treatment works well with stimulants for ADHD and also Selective Serotonin Reuptake Inhibitors (SSRIs) for anxiety.

Prescribers must consider medications not only for symptoms of associated psychopathology but also as targeted treatments that have the potential to reverse the neurobiological abnormalities and should be considered as a part of an individualized therapeutic program with behavioral and educational interventions.

### General Principles in Using Pharmacological Treatment in ASD

Frequently, identification and management of psychiatric issues can be complex, especially for those with limited language repertoire, low cognitive function, and those experiencing uncertain symptoms. Diagnostic overshadowing is common (failure to identify other conditions in the presence of a certain diagnosis). A high level of clinical suspicion for co-occurring mental health conditions is required for children and adolescents with communication challenges. Managing clinicians should obtain information from the child, when possible, family and other providers including teachers and therapists. Environmental changes and lack of skills can be the source of undesired behaviors and should be considered in the plan of care.

Pharmacological interventions are sometimes indicated and may facilitate their participation in therapy and enhance their daily functioning. The principles used for psychopharmacological management are the same for children with ASD as for those with typical development. However, prescribers should keep in mind that children with ASD tend to be more sensitive to medication effects and more likely to have adverse effects than children without ASD. Therefore, pharmacological treatment should be started at lower doses, and adjusted more slowly than in neurotypical children. Obtaining objective symptom measures from different sources before and after the intervention is key to objectively evaluate the response of treatment in different settings.

### Serotoninergic Medications

Serotoninergic medications regulate the levels of serotonin which is a key messenger specially involved in the gastrointestinal, cardiovascular, and the central nervous system (CNS). The serotonin level has been reported to be elevated in the autistic population, and it has been theorized that serotonin dysregulation is associated with symptoms frequently seen in autistic individuals ranging from repetitive behaviors to anxiety. PET studies have demonstrated that young children (under 5 years old) with ASD have lower levels of serotonin in the CSF [31]. Studies of lymphoblastoid cell lines in patients with ASD compared to controls have demonstrated a deficit of enzymes that convert tryptophan to serotonin [32]. These studies suggested that those with ASD would benefit from treatment with an SSRI to stimulate neurogenesis and neuroprotection [33]. There are three different groups of medications that influence the serotonin levels: the SSRIs, SNRIs (serotonin-norepinephrine reuptake inhibitors), and tricyclic antidepressants. The SSRIs are one of the most commonly prescribed medications for autistic individuals to treat anxiety, mood issues, and irritability. However, results of available clinical trials have been inconsistent in the benefits of SSRI’s for improving aggression and the core symptoms of ASD [34].

A retrospective study of children with FXS (ages 12 to 50 months) demonstrated improvement in the trajectory of both receptive and expressive language measures on the Mullen Scales of Early Learning (MSEL) in those treated with low-dose sertraline vs those who did not receive sertraline [35]. These results led to a controlled trial for 6 months of sertraline in children ages 2 to 6 with FXS (60% also had ASD) treated clinically with low dose sertraline (2.5 to 5.0 mg/day) [36]. Those treated with sertraline demonstrated greater improvement in motor and visual subtests and the Cognitive T score on the MSEL compared to those on placebo. In the children with both FXS and ASD, there was also significant improvement on the Expressive Language subscale compared to placebo [36]. In the same controlled trial, a passive visual eye tracking measure of receptive vocabulary was also significantly improved in those treated with sertraline compared to placebo [37]. These studies suggest that young children with FXS both with and without ASD benefit from low-dose sertraline treatment. However, a similar study in young children ages 2 to 6 with idiopathic ASD (without FXS) treated with low-dose sertraline did not demonstrate a benefit of sertraline compared to placebo [38]. Therefore, the genetic subtype of ASD makes a difference in response to treatment and all children diagnosed with ASD must have genetic testing including Fragile X DNA testing and a CGH array for starters and subsequent whole exome sequencing (WES) or whole genome sequencing (WGS) if the initial studies are negative [39].

### Atypical Antipsychotics

There are two medications approved by the FDA for the treatment of irritability associated with ASD: risperidone, approved for children older than 5 years of age [40], and aripiprazole, approved for children 6 to 17 years of age [41]; clinical trials found them to be effective in reducing irritability and, to a lesser degree, repetitive behaviors. These two atypical antipsychotic medications have affinity for dopamine, 5-HT, alpha-adrenergic, and histaminergic receptors in the brain. They also share similar safety profiles; the most common side effects include fatigue, increased appetite, GI symptoms, hyperprolactinemia, weight gain, and sedation, and less commonly activation including restlessness and akathisia. They are also linked to more serious side effects including dyslipidemia, hyperglycemia, metabolic syndrome, and extrapyramidal symptoms or drug-induced movement disorders. Therefore, close clinical and laboratory monitoring is recommended. Given that the efficacy and safety of these medications have not been established for the long-term treatment of irritability in autistic individuals, it is important to periodically re-evaluate the need for continuation of treatment. Since the development of atypical antipsychotics, the use of the conventional antipsychotics has been reserved for more severe cases refractory to the newer generation medications, due to the narrower safety profile and greater incidence of adverse reactions including extrapyramidal symptoms such as tardive dyskinesia with conventional antipsychotics.

### Stimulant Medications

Stimulants are usually the first line of treatment to treat co-occurring attention deficit and hyperactivity disorder (ADHD) as they present with a rapid clinical effect and there is enough data supporting their use and safety. Approximately half of autistic children also meet criteria for ADHD [42], but prevalence widely varies based on samples [8, 43–47]. Treating co-occurring ADHD symptoms in autistic individuals should focus on improvement in enhancing their daily function in multiple settings, including learning, and hopefully long-term functional outcomes improving associated symptoms causing impairment in the academic setting, peer relationships, and emotional regulation, which are also key predictors and mediators of functional difficulties in adulthood. Before starting a patient on a regimen, the prescribing clinician should assess the potential risks for pharmacotherapy by obtaining a complete past medical history, family history, and a physical examination with a specific focus on the cardiovascular system. It is important to obtain pretreatment baseline information and a close follow up to objectively evaluate the impact of common side effects associated with pharmacotherapy for ADHD (i.e., appetite changes, hypertension, weight loss, sleep disturbances, headaches, abdominal pain). Baseline sleep problems do not appear to predict stimulant-related sleep problems and may improve with stimulant therapy [48]. Adolescent patients should be assessed for substance use or abuse prior to starting treatment.

There are two main stimulant families: the amphetamines are usually slightly more efficacious than the methylphenidate derivates which are usually better tolerated [49]. In a systematic review and network meta-analysis that included 81 published and unpublished randomized trials in > 10,000 neurotypical children, amphetamines were slightly more efficacious than methylphenidate in reducing clinician-rated core symptoms of ADHD at approximately 12 weeks; however, amphetamines were less tolerable than placebo and methylphenidate was better tolerated than amphetamines [49]. Specific systematic review of four-crossover trials in autistic children (113 participants) age 5 to 13 years found low-quality evidence that short-term treatment with methylphenidate may improve hyperactivity and inattention in children with ASD, and the only significant adverse side effect was reduced appetite as rated by parents; however, there was no evidence of impact on core ASD symptoms or improvement in social interaction [50]. In the largest crossover trial, approximately 50% of children with ASD responded to methylphenidate based on the hyperactivity subscale of the Aberrant Behavior Checklist (ABC); the effect size ranged from 0.20 to 0.54, depending upon dose and rater, with greater improvement at higher doses; then, this modest effect supports that Methylphenidate exerts a lower effect on primary ADHD symptoms in individuals with ASD compared to those in the neurotypical population. Six of 66 children in the double-blind phase (9.1%;) discontinued treatment due to adverse effects, including irritability, repetitive behaviors, tics, insomnia, and reduced appetite [51].

Treatment failure is defined by lack of satisfactory improvement in core symptoms of ADHD at the maximum dose or the occurrence of intolerable adverse effects. At least half of the children who presented with an inadequate response or side effects to a certain medication may respond well to another one. For those children failing to respond to two different medications, the prescriber should evaluate other causes for the limited therapeutic response including (1) the presence of comorbid psychiatric diagnosis, (2) unrealistic expectations about the expected clinical response, (3) misuse or medication diversion, and (4) lack of adherence to the regimen. Children on stable maintenance dose should be followed every 6 months to monitor side effects and evaluate clinical response.

### Alpha-2-adrenergic Agonists

There is also evidence about the use of alpha 2 agonists to improve core ADHD symptoms, but alpha-2-adrenergic agonists (i.e., guanfacine and clonidine) are frequently used in children under 5 year old with ADHD or hyperarousal, cases with poor response to a trial of stimulants, or selective norepinephrine reuptake inhibitors, have unacceptable side effects, or have significant co-occurring conditions (i.e., sleep issues). However, studies of alpha-2-agonists in ASD are limited and have small sample sizes. Guanfacine has been reported to be safe and effective in the treatment of hyperactivity and impulsiveness in children with ASD [52, 53]. The most common side effects of guanfacine include sedation, constipation, irritability, and aggression. A small crossover study has also suggested positive effects of clonidine in ASD including decreased irritability, stereotypy, hyperactivity, inappropriate speech, and hyperarousal behaviors [54].

Data from randomized trials, systematic reviews, and meta-analyses show that atomoxetine and alpha-2-adrenergic agonists are more effective than placebo in reducing the core symptoms of ADHD, but as a class, they are less effective than stimulants [49, 55, 56]. Similarly, it is key to obtain objective targeted symptom measures at baseline and during treatment to objectively evaluate the response to treatment in different settings.

In a recent review of nine controlled trials of 430 children with ASD comparing the response between methylphenidate, atomoxetine, and guanfacine, methylphenidate and atomoxetine had superior effects than placebo in addressing ADHD symptoms; however, the response for hyperactivity symptoms was less than observed in neurotypical populations with both medications [57]. Worse treatment outcomes were associated with individuals with lower cognitive functioning.

### Melatonin

Sleep issues are frequently reported in children with ASD potentially affecting their behavior, daily functioning, and family life. There is some evidence suggesting that low melatonin levels affect the circadian rhythm in autistic children [58]. In cases where behavioral and environmental sleep interventions have been implemented with limited response, clinicians may recommend the use of melatonin which is usually well tolerated and has a low incidence of side effects [59]. There is increasing evidence for the use of prolonged-release melatonin in autistic individuals with limited response to regular release formulations [60]. Melatonin is an over-the-counter product that is not regulated by the FDA. When parents/caregivers purchase melatonin, they should seek a formulation that contains melatonin as the only active ingredient.

### N-acetylcysteine

N-acetylcysteine (NAC) is another antioxidant that can be purchased over the counter (OTC), and it can improve the imbalance of excitation: inhibition (E:I) that is seen in some forms of ASD [61]. NAC works by two mechanisms to lower the E:I imbalance; it lowers glutamatergic neurotransmission, and the cysteine leads to an increase in glutathione synthesis which is an important antioxidant. Cysteine is also oxidized to cystine, which further helps to reduce glutamatergic neurotransmission [62]. Hardan and colleagues carried out a controlled trial of escalating doses of NAC from 900 mg once a day for 4 weeks, increasing to bi-daily dosing for 4 weeks and then tri-daily dosing for the last 4 weeks compared to placebo. They randomized 33 subjects with ASD ages 3.2 to 10.7 years and after 12 weeks of treatment they found significant improvement on their primary outcome measure, irritability subscale on the ABC (*p* < 0.001) for patients treated with NAC compared to placebo. Additional improvements were seen in stereotypic behaviors with significance reached on the RBS-S Stereotypies subscale (*p* < 0.014) and the SRS Autism Mannerisms subscale (*p* < 0.045) for those treated with NAC vs placebo [62]. NAC was well tolerated although an occasional patient did not like the taste or had minimal gastrointestinal side-effects.

### Dietary Supplements

Sulforaphane is a naturally occurring isothiocyanate (found in broccoli and other cruciferous vegetables) [63–65]. Sulforaphane is an antioxidant, anti-inflammatory, and mitochondrial protective agent that has been studied in several animal models and humans with neurodegenerative and neurodevelopmental disorders [66]. Sulforaphane is a sulfur-rich dietary phytochemical which can penetrate the blood brain barrier, and it subsequently induces the nuclear factor erythroid 2 related factor 2 (*Nrf2*) signaling cascade that stimulates the expression of more than 200 genes that are antioxidants and involved in detoxification and neuroprotection in the CNS [67]. The effect leads to reduction of superoxide and other reactive oxygen species (ROS), upregulation of the proteozome system to digest unfolded or misfolded proteins, enhancement of autophagy, inhibition of pro-inflammatory cytokines, protection from heme toxicity, and defense of neuronal cells from Aβ_42_-mediated cytotoxicity.

There have been a few studies in patients with ASD [68, 69] including a controlled trial of young men ages 13 to 27 with moderate to severe ASD treated with sulforaphane (*n* = 29) compared to placebo (*n* = 15) for 18 weeks. Significant improvements were seen on the Aberrant Behavior Checklist (ABC), the Clinical Global Improvement Scale (CGI-I), and the Social Responsiveness Scale 2 (SRS) [69]. This positive trial lead to a more detailed study in children with ASD, a randomized controlled trial of sulforaphane lasting 15 weeks followed by an open label trial for another 15 weeks in 57 children ages 3 to 12 years [70]. Although the primary outcome measure, the Ohio Autism Clinical Impressions Scale, did not improve significantly in those on sulforaphane, a secondary measure, the ABC, did significantly improve on sulforaphane vs placebo but the SRS did not. In addition, there were significant improvements in the biomarkers including the glutathione redox status, mitochondrial respiration, inflammatory markers, and heat shock proteins on sulforaphane vs placebo, and these improvements correlated with improvements on the ABC. They utilized a commercial product of sulforaphane called Avmacol made by Nutrimax with a tablet dose of 2 to 8 tablets per day depending on the weight of the child (equivalent to 2.2 μmol/kg/day). There were no significant adverse events and the supplement was well tolerated.

Other antioxidants have been studied in ASD including omega-3 fatty acids [71, 72] with mixed results, and these antioxidants promote glutathione recycling by facilitating the conversion of oxidized glutathione into reduced glutathione. A more recent study was carried out by Mazahery et al. [73] in 111 children with ASD ages 2.5 to 8 years, and they were randomized to placebo, Vitamin D 2000 IU/day, or omega-3 722 mg/day or both interventions for 1 year of treatment. Seventy-three patients completed a year of therapy, and those on both treatments had a significant reduction in their primary outcome measure, irritability on the ABC subscale (*p* < 0.001) compared to placebo, and those treated with vitamin D alone also had a reduction in irritability also compared to placebo (*p* < 0.45) [73]. These studies suggest that antioxidants may be a helpful ancillary treatment in some patients with ASD, although biomarkers of oxidative stress would be helpful to assess in further studies to better identify those who would benefit from this treatment.

## Emerging Targeted Treatments with a Possible Role in ASD

### Oxytocin

Oxytocin (OXT) is a neuropeptide synthesized in the hypothalamus that plays a critical role in social functioning. Extant literature has shown that OXT enhances social processing in typically developing adults (enhanced eye contact, better emotion recognition in faces) immediately after its administration [74]. There have been generally positive results of OXT in adults with ASD, with trials showing improvements in repetitive behaviors, social reciprocity, and emotion recognition [75–77]. However, all these trials studied only short-term benefits (within a few weeks) of OXT administration. A recent randomised, placebo-controlled, double-blind study in adults with ASD showed improvements in self-reported repetitive behaviors and positive mood at 1 year post treatment after an initial 4 weeks of oxytocin treatment [78]. However, in this same study, there were no significant treatment benefits for social responsiveness with OXT [78]. Another recent study in young adults with ASD also did not demonstrate any immediate benefits of OXT on empathy and social perception [79].

Results of OXT studies in children have overall been more equivocal with mixed results. Although 4 studies showed positive short-term results of OXT administration on social responsiveness (following 4 or 5 weeks of OXT administration) [80–83], another 2 studies did not demonstrate any OXT specific improvements in social responsiveness or repetitive behavior in children with ASD [84, 85]. A recent randomized controlled trial (RCT) however did not show any significant effects between the OXT and placebo group in aberrant behavior, social communication, or cognition [86]. At the neural networks level, it has been shown that intranasal OXT leads to increased activation in the brain regions known to be involved in perceiving and thinking about social-emotional information and enhances effective connectivity between nodes of the brain’s reward and socioemotional processing systems [87, 88]. There were no noted side effects in these studies on children with ASD, thus far, although animal studies have raised the possibility of increased basal OXT levels with long-term OXT administration; the clinical effects of this being unclear. Of pertinence, there remains a lack of conclusive evidence for the long-term beneficial effects of OXT in addressing the core symptoms of autism [89]. Given that the vast majority of studies in children utilize parent-reported outcome measures of social and behavioral symptoms, inherent limitations of bias in reporting even in placebo-controlled trials are likely to come into play. Another important consideration is whether the gains that are seen with OXT administration in the experimental setting translate to real life and this is also unclear. The role of OXT thus far has been limited to its immediate effect after administration and hence is not a single treatment option for ASD. Nonetheless, as illustrated by a recent meta-analysis, there does seem to be overall beneficial effects of OXT on social symptoms of ASD, although this review included both children and adults [90]. There is also some promising research looking at the role of OXT in combination with other treatment modalities including behavior therapy and probiotics, with clinical trials in this area ongoing [91, 92]. It is also likely that the effects of OXT in ASD are modulated by age, gender, and possibly genetic factors [77, 79, 90]. As such, although it holds much promise, the use of OXT in individuals with ASD is currently not a mainstream treatment.

### Bumetanide

Bumetanide is a well-established loop diuretic that works by inhibiting sodium–potassium-chloride co-transporters, namely, NKCC1 and NKCC2. Bumetanide has been purported as a potential treatment in autism due to its inherent chloride-related antagonist effects which is linked to GABA-ergic inhibition [93]. Bumetanide has been shown to reduce broad ASD symptomatology in children following a 3-month treatment course in 2 placebo-controlled randomized controlled trials [94, 95]. Both of these trials used outcomes that are screening tools for ASD, namely, the SRS and the Childhood Autism Rating Scale (CARS). Another open-label trial of 6 children with severe ASD and intellectual disability showed parent-reported improvement in communicative abilities of all children after 3 months of bumetanide [96]. However, a recent double-blind, placebo-controlled, phase 2 superiority trial in children with ASD without severe intellectual disability did not show any treatment benefits on the core symptoms of ASD as measured on the SRS-2 [97]. It did show treatment benefits on the repetitive behavior scale, with no major adverse effects. Another study has suggested possible combined effects of bumetanide with ABA therapy in improving ASD symptoms on the CARS, although this was not a randomized controlled trial [98]. There are 2 phase 3 clinical studies ongoing now, which may shed further information on the potential benefits of bumetanide in ASD [99]. There is some functional-MRI-based evidence suggesting that bumetanide reduces the exaggerated amygdala activation to eye contact in individuals with ASD and contributes to increased eye-gaze time with biological stimuli and better emotional face perception [100, 101]. Regardless, based on current literature, there is inconclusive evidence for the role of bumetanide in addressing the core symptoms of ASD [102, 103].

### Metformin

Targeted treatments that reverse known neurobiological abnormalities in subgroups of ASD where there is also animal data to demonstrate benefit have emerged in the last decade for subgroups of ASD. The subgroup of ASD that is leading the way in targeted treatments is FXS, the most common single gene cause of ASD. In addition, post mortem studies have shown that FMRP, the protein that is missing or deficient in FXS, is also deficient in the brain in patients with idiopathic ASD without a fragile X mutation [104, 105]. Therefore, FXS is a model for targeted treatments in other subtypes of ASD and treatments that work well in FXS may also be beneficial for other forms of ASD. So we will describe some of the targeted treatment studies with compounds that are available currently, although not FDA approved for FXS nor ASD.

Animal studies in FXS have demonstrated a hyperactive insulin receptor and up-regulation of the mammalian target of rapamycin complex 1 (mTORC1) and mitogen-activated protein kinase/extracellular signal-related kinases (MAPK/ERK) signaling pathways, as well as elevation of MMP-9 levels in the absence of FMRP, the protein produced by the *FMR1* gene [106–108]. Metformin is a bi-guanide that is a primary treatment for type 2 diabetes, but it can also reduce the appetite in individuals with obesity. Therefore, studies of metformin were first carried out in patients with FXS who demonstrated obesity, often with the Prader-Willi-phenotype of FXS [109]. In a handful of patients with FXS treated clinically with metformin between the ages of 4 and 60 years old, there was improvement in overeating but also on the ABC subscales of irritability, aggression, and social avoidance [109]. Parents also stated that they saw improvement in the expressive language abilities in conversation. The potential language improvements are currently being studied in a controlled trial of metformin occurring over 3 sites, 2 in Canada (Edmonton and Montreal), and one site in the USA at the MIND Institute funded by the Azrieli Foundation (NCT03479476, NCT03862950). Patients ages 6 to 45 are recruited into a randomized controlled trial lasting 4 months with outcome measures including the Expressive Language Sampling as the primary outcome but also event related potentials, eye tracking, NIH toolbox, and other behavioral measures are assessed. The results will be available in 2022. Additional open label studies have been carried out with metformin including one in children ages 2 to 7 years old with FXS, and improvements were seen in behavior and development on the MSEL [110]. Individual case studies have shown that macroorchidism did not develop in boy who started metformin clinically before puberty [111] and two adults with FXS improved their IQ when using metformin for over one year [112].

### Lovastatin

Lovastatin is a commonly used statin that lowers cholesterol levels, but it does this by inhibiting 3-hydroxy-3methylglutaryl coenzyme A (3HMG-CoA) reductase, and it is FDA approved for lowering hypercholesterolemia or hyperlipidemia in children and adults. This action lowers the excessive protein production of the MEK-ERK pathway which are elevated in FXS. Studies of lovastatin treatment in the FXS knock out (KO) mouse rescued excess protein synthesis and also epilepsy [113]. These animal studies stimulated FXS patient trials. The one controlled trial included 32 children with FXS between 10 to 17 years treated in a RCT for 20 weeks with a dose of 10 to 40 mg a day as tolerated [114]. In addition, the patients all received Parent Implemented Language Intervention (PILI) [115] delivered by distance video teleconferencing over 12 weeks by a speech and language therapist with 4 sessions per week. Parents were taught a set of language facilitation techniques that were utilized with shared story telling sessions with their child. The main outcome measures were the number of utterances and new words utilized in addition to additional language scales, behavioral measures (ABC), and the CGI-I. So this study compared the combined effects of lovastatin plus PILI to PILI alone with placebo. Remarkably, there was significant improvements from baseline in both groups, but the outcomes were the same in both groups; that is, PILI alone had as much improvement as lovastatin plus PILI demonstrating the power of intensive language intervention [114].

### Cannabidiol

Cannabidiol (CBD) is a phytocannabinoid found in *Cannabis sativa*, marijuana. Although there are hundreds of phytocannabinoids in marijuana, CBD is the second most common one after delta-9-tetrahydrocannabinol (THC) which has psychotropic properties. Marijuana has been used for 8000 years in India, China, and Middle East for fiber and medicinal properties; then introduced to Europe in early nineteenth century by Napoleon’s army returning from Egypt and then to Britain for medical use by a surgeon who served in India. CBD is the non-psychotropic component of marijuana, and there are numerous therapeutic effects of this drug including treatment of anxiety, pain, nausea, and motor deficits including the tremor in Parkinson’s disease [116]. CBD has both neuromodulatory and neuroprotective effects through a number of mechanisms including blocking neuroinflammation and potentiating anti-inflammatory pathways, improving mitochondrial function, GABA_A_ agonist potentiation, stimulation of 5HT_1A_ receptors, and enhancing levels of anandamide (AEA) [116–119].

The endocannabinoid system has two receptors CB_1_ found primarily in the CNS and CB_2_ found throughout the body and the immune system. The primary endogenous ligands for CB_1_ and CB_2_ receptors are called endocannabinoids (ECs) and include anandamide AEA and 2-arachidonoylglycerol (2-AG). The ECs modulate synaptic transmission throughout the CNS, yielding widespread influence on cognition and behavior. The ECs are synthesized and released from post-synaptic membrane-bound phospholipids in response to neuronal signaling and act as retrograde signaling molecules across the synaptic cleft to stimulate CB_1_ receptors on the presynaptic terminal, and they can inhibit neurotransmitter release from the presynaptic terminal. Enzymes that function in synthesizing 2-AG include phospholipase C and diacylglycerol lipase (DAGL).

CBD has also been shown to act as a positive allosteric modulator at GABA_A_ receptors, and controlled trials have shown that CBD in the form of Epidiolex is an effective anticonvulsant in Dravet syndrome and Lennox-Gastaut syndrome [120]. CBD’s ability to enhance endocannabinoid levels and facilitate GABAergic transmission may serve to improve the balance in inhibitory and excitatory transmission and help restore neuronal function and synaptic plasticity in patients with ASD and FXS even when there is no epilepsy. Animal models of both FXS and ASD have shown benefits when treated with CBD [121, 122]. Studies of individuals with ASD treated with CBD and open label trials of CBD are reviewed by Nezgovorva et al. [123]; however, the preparations studied have both CBD and variable levels of THC, although in general, benefits were seen in irritability, sleep disorders, tantrums, and anxiety. Currently, studies of cannabidavarin (CBDV) are taking place in ASD and CBDV has also been helpful in animal models of ASD [123].

Recently, the development of a topical CBD that is manufactured so that there is no THC has facilitated studies in both ASD and in FXS. The BRIGHT study was an open label study of children ages 3 to 17 with ASD lasting 14 weeks, and benefits were seen in most outcome measures including the ABC and measures of anxiety [124]. Currently, a controlled trial of this topical CBD called Zyn002 is taking place in children with ASD. Another recent randomized controlled trial (RCT) looking at an oral preparation of CBD in children and young adults with ASD demonstrated positive improvements in behavior and social communication with CBD [125].

Huessler et al. [126] carried out an open label trial of Zyn002 in Australia for children with FXS of ages 3–17 years old with doses of the transdermal CBD at doses 250 mg bi-daily for 12 weeks (ACTRN12617000150347). Both the primary outcome, the Anxiety Mood and Depression (ADAMS) scale and the secondary measures including the ABC, demonstrated efficacy. Subsequently, a multicenter controlled trial of over 200 children with FXS was carried out and efficacy was seen in only those children with > 90% methylation with FXS on the primary outcome measure of the Social Avoidance subscale of the ABC_FX_, a scale that has been developed for FXS modified from the ABC (Berry-Kravis et al. 2022 under review Sci Trans Medicine). Currently, the FDA has not approved Zyn002 for general use, but an additional multicenter controlled trial is now taking place to win this approval. It is very likely that the current controlled trials taking place for ASD and FXS will show efficacy for subgroups for both disorders, and subsequently, CBD will be more broadly utilized.

### Arbaclofen

Arbaclofen, also called STX209, is a selective ɣ-aminobutyric acid type B receptor agonist that is the R-enantiomer of racemic baclofen. For many subtypes of ASD, there is a GABA_B_ deficit and arbaclofen has rescued the behavioral deficits including social deficits in the mouse models of idiopathic ASD [127], deletion of 16p11.2 [128], and FXS [129]. There are 3 pathways that are improved with arbaclofen: Stimulation of presynaptic GABA_B_ receptors inhibits glutamate release thereby lowering the mGluR5 pathway. Stimulation of GABA receptors also improves inhibition that is down-regulated in many forms of ASD and arbaclofen also enhances K channel activation which can also be down-regulated in many forms of ASD [130]. The promising mouse studies led to human trials in FXS [131] that initially showed improvements in those with ASD plus FXS leading to phase 3 trials in FXS [132]. However, the adult studies of FXS did not demonstrate efficacy and the pediatric trials did not reach significance for the primary outcome measure, but did show limited improvements in secondary measures including the Parenting Stress Index because of lowered irritability in the children [132].

Both open label studies in idiopathic ASD and in those with a 16p11.2 deletion have been carried out with positive behavioral benefits. A controlled trial with idiopathic ASD has also been started but not yet reported. Arbaclofen has been well tolerated even at higher doses up to 15 mg tri-daily so it is likely that further studies will be carried out both in ASD and in subtypes of ASD including FXS.

### Trofinetide

Insulin like growth factor 1 (IGF1) is considered an emerging treatment for ASD in animal and cellular models in ASD [133–136]. Trofinetide is an analogue of the amino-terminal tripeptide of IGF1, and it has been studied in patient groups of ASD subtypes. Trofinetide is glycyl-L-2-methylprolyl-L-glutamic acid, and it was studied in a controlled phase 2 trial in 82 children with Rett syndrome ages 5 to 15 years, and significant benefit was found in the high-dose group (200 mg/kg/day) compared to placebo [137]. Significant benefits were seen in several measures including the Rett Syndrome Behavior Scale, the Rett Syndrome Clinician Rating Scale, and a visual analogue scale. This report led to a multicenter phase 3 controlled trial in Rett syndrome which is ongoing currently.

Trofinetide has also been studied in a 28-day controlled trial in adolescent and adult patients with FXS [138]. Patients were randomized to trofinetide 35 mg/kg/day, 70 mg/kg/day, or placebo. Results demonstrated that the 70 mg/kg/day was significantly beneficial compared to placebo with a permutation test utilizing the primary components of the Fragile X Syndrome Rating Scale, a Fragile X Specific Domain Scale on a visual analogue format, and the ABC_FX._

In the fragile X knockout (KO), mouse studies trofinetide had several positive effects at a dose of 100 mg/kg/day yielding insight as to why it is beneficial in FXS [139]. The KO mouse was deficient in IGF1 in the brain, and this was normalized with trofinetide treatment for 28 days. Improvements in dendritic spine abnormalities, astrogliosis, neuroinflammation, glial activation, and downregulation of the MEK-ERK and PI3K-mTOR pathways were seen with trofinetide treatment leading to improvements in behavior and morphology of FXS [139]. Clearly, trofinetide is a treatment that improves multiple pathways that are dysregulated in more than one subtype of ASD and further studies at optimal doses will be carried out and some are currently taking place.

### Phosphordiasterase 4D Inhibitors

It has been known for many years that cAMP, an important energy compound for improving synaptic connections, is down regulated in FXS [140]. Recent animal studies have shown that an inhibitor of cAMP breakdown called a phosphordiasterase 4DE inhibitor can rescue features of FXS in the KO mouse model and *Drosophila* model and can raise the cAMP levels to normal [141, 142]. These studies led to patient trials of a PDE4D inhibitor called BPN14770, and an exciting study was recently published, a randomized controlled trial in 30 adult males with FXS that demonstrated improvements not only in behavior but also in the primary outcome measure, the NIH toolbox, and secondary measures after only 12 weeks of treatment [143]. This is the first treatment of FXS that demonstrated improvements in cognition, specifically in Oral Reading Recognition, Picture Vocabulary and Cognition Crystallized Composite Score in the NIH toolbox that has been modified for use in those with ID. The caregivers also used the Visual Analog Caregiver Rating Scales and demonstrated improvement in language and daily functioning. Families are excited that this is the first of hopefully many new medications that can reverse cognitive deficits and further controlled trials are in the planning stages.

### Anavex 2–73

Anavex 2–73 (AV 2–73; Blarcamesine) is a sigma 1 receptor agonist that works between the endoplasmic reticulum and the mitochondrial membrane to normalize calcium dysregulation, oxidative stress, and mitochondrial dysfunction which is seen in many forms of ASD. It has demonstrated significant benefits in the KO mouse model of FXS where multiple behaviors were improved and deficient brain derived neurotropic factor (BDNF) levels were normalized [144]. In addition, Kaufman et al. [145] also reported significant benefits in the Rett syndrome mouse model with a rescue of behavior and BDNF levels and this work lead to patient studies in Rett syndrome that have demonstrated efficacy in a controlled trial (Anavex Life Sciences press release 2021). AV2-73 also has beneficial effects in neurodegenerative disorders because of improvement in proteostasis, autophagy, oxidative stress, prevention of protein aggregates, and improvement in mitochondrial function leading to benefits in Alzheimer’ disease and Parkinson’s disease dementia [146–148]. Significant potential exists for AV2-73 to improve symptoms in Fragile X-associated Tremor Ataxia (FXTAS), a neurodegenerative disease seen in approximately 40% of older carriers of the fragile X premutation, because calcium dysregulation, mitochondrial dysfunction, proteostasis, and aggregations of proteins causing inclusions occur in FXTAS [149].

### Gene Therapy

Although this therapy is not available for clinicians to utilize in their patients, exciting research studies particularly after the advances in CRISPR/Cas9 technology have become available. The possibility of introducing a normal gene or protein into the CNS to treat ASD or other neurodevelopmental disorders where the mutation is known is exciting. Another example of gene therapy is the introduction of antisense oligonucleotides (ASOs) to silence RNA or gene products that are deleterious. In Angelman syndrome, where the maternal copy of UBE3A is mutated or absent, ASOs have been utilized to activate the paternal copy of UBE3A in the CNS to compensate for the missing maternal copy. Recently, a controlled trial of GTX-102, an ASO, was tried in 5 individuals with Angelman syndrome ages 5 to 15 years old. The protocol involved the intrathecal injection of GTX-102 at increasing doses once monthly for 4 months. However, an adverse effect of leg weakness was seen at the higher doses leading to an inability to walk in two patients. This was found to be related to inflammation at the level where the LP was carried out so these patients were treated with anti-inflammatories with resolution of this side effect. The future is bright for further gene therapy interventions in ASD and other neurodevelopmental disorders.

## Conclusion

The current evidence-based management of ASD in children relies primarily on behavioral interventions to address the core symptoms of the condition. The role of pharmacological treatments currently is primarily to address co-morbid conditions associated with ASD and increases with age. These medications including anti-psychotic agents and stimulant medications are important in the clinical management of patients with ASD. However, the emergence of targeted treatments for subgroups of ASD where the genes responsible for the ASD are known and the neurobiology and potential targeted treatments have been studied to reverse the neurobiological abnormalities at least in the animal models has led to several recent achievements in patients as described here. Of note is that there are commonalities among disorders causing ASD that suggest that a targeted treatment for one disorder will be beneficial for other disorders. For instance, GABA deficits are seen in many forms of ASD and medications that are agonists for the GABA system such as CBD are likely to be helpful for many subtypes of ASD as described above. Mitochondrial dysfunction is associated with many forms of ASD, so medications that will improve mitochondrial dysfunction are likely to be helpful for many subtypes of ASD [150]. The promise of gene therapy is becoming a reality for many disorders such as Duchene Muscular Dystrophy, Spinal Muscular Atrophy, and even Angelman Syndrome because of CRISPR/Cas 9 technology so in the next few years, many additional forms of ASD will be treated with gene therapy. Until then, some of the treatments outlined here can be tried and more will become available in the near future.

## Supplementary Information

Below is the link to the electronic supplementary material.

Supplementary file1 (PDF 436 kb)

Supplementary file2 (PDF 435 kb)

Supplementary file3 (PDF 508 kb)

Supplementary file4 (PDF 433 kb)
